# Royal Medical and Chirurgical Society

**Published:** 1897-04-03

**Authors:** 


### ROYAL MEDICAL AND CHIRURGICAL SOCIETY.

At the last meeting, Dr. Duggan read a paper on "The Parasite of the Malarial Fevers at Sierra Leone." During two years' residence he had examined blood from the finger in nearly 400 cases. After describing the symptoms met with, he said that in all cases he found the parasite in blood from the finger. He then described the appearances observed, which were similar to those described by Marchiafava as being found in the summer-autumn fevers of Rome. Morphologically, the author of the paper was not able to detect any difference between Marchiafava's parasite and that of Sierra Leone. In the discussion, Dr. Thin said that the fevers on the West Coast of Africa were extremely fatal as compared with those in the South of Europe. This, he thought, was due to a plurality of parasites. As illustrating the dangers to Europeans, he mentioned an instance in which four out of five artisans died from fever almost immediately after arrival in the country; one of them being attacked on the second day (which showed an unusually short period of incubation) and dying on the fourth. It was remarkable what a protective influence the regular administration of quinine had in regard to this disease. Dr. Manson insisted on the importance of microscopic examination of the blood in such cases for purposes of diagnosis, for many cases which were now known to be cases of severe malaria would formerly have been returned as cases of sunstroke.

